# Relationship, evolutionary fate and function of two maize co-orthologs of rice *GW2 *associated with kernel size and weight

**DOI:** 10.1186/1471-2229-10-143

**Published:** 2010-07-14

**Authors:** Qing Li, Lin Li, Xiaohong Yang, Marilyn L Warburton, Guanghong Bai, Jingrui Dai, Jiansheng Li, Jianbing Yan

**Affiliations:** 1 National Maize Improvement Center of China, Key Laboratory of Crop Genomics and Genetic Improvement (Ministry of Agriculture), China Agricultural University, 100193 Beijing, China; 2 USDA-ARS Corn Host Plant Resistance Research Unit Box 9555 Mississippi State, MS 39762; 3 College of Agriculture, Xinjiang Agricultural University, Urumqi, 830052 Xinjiang, China; 4 International Maize and Wheat Improvement Center (CIMMYT), Apdo. Postal 6-641, 06600 Mexico, D.F., Mexico

## Abstract

**Background:**

In rice, the *GW2 *gene, found on chromosome 2, controls grain width and weight. Two homologs of this gene, *ZmGW2-CHR4 *and *ZmGW2-CHR5*, have been found in maize. In this study, we investigated the relationship, evolutionary fate and putative function of these two maize genes.

**Results:**

The two genes are located on duplicated maize chromosomal regions that show co-orthologous relationships with the rice region containing *GW2*. *ZmGW2-CHR5 *is more closely related to the sorghum counterpart than to *ZmGW2-CHR4*. Sequence comparisons between the two genes in eight diverse maize inbred lines revealed that the functional protein domain of both genes is completely conserved, with no non-synonymous polymorphisms identified. This suggests that both genes may have conserved functions, a hypothesis that was further confirmed through linkage, association, and expression analyses. Linkage analysis showed that *ZmGW2-CHR4 *is located within a consistent quantitative trait locus (QTL) for one-hundred kernel weight (HKW). Association analysis with a diverse panel of 121 maize inbred lines identified one single nucleotide polymorphism (SNP) in the promoter region of *ZmGW2-CHR4 *that was significantly associated with kernel width (KW) and HKW across all three field experiments examined in this study. SNPs or insertion/deletion polymorphisms (InDels) in other regions of *ZmGW2-CHR4 *and *ZmGW2-CHR5 *were also found to be significantly associated with at least one of the four yield-related traits (kernel length (KL), kernel thickness (KT), KW and HKW). None of the polymorphisms in either maize gene are similar to each other or to the 1 bp InDel causing phenotypic variation in rice. Expression levels of both maize genes vary over ear and kernel developmental stages, and the expression level of *ZmGW2-CHR4 *is significantly negatively correlated with KW.

**Conclusions:**

The sequence, linkage, association and expression analyses collectively showed that the two maize genes represent chromosomal duplicates, both of which function to control some of the phenotypic variation for kernel size and weight in maize, as does their counterpart in rice. However, the different polymorphisms identified in the two maize genes and in the rice gene indicate that they may cause phenotypic variation through different mechanisms.

## Background

The genetic improvement of grain yield in major cereals has traditionally been one of the most important contributions to an increased global food supply. Gains via phenotypic selection have been steady but slow over the years, and the ever-growing world population makes it necessary to increase the rate of gain in grain yields over what has been achieved in the past. Grain size and weight are important components of grain yield, but the genetic basis of these traits in maize (*Zea mays*), one of the most important global food staples, is insufficiently understood. To date, several loci have been shown to affect maize kernel development through mutant analysis [1-5], but only one gene (*GS3*) has been found to affect kernel size and weight in a natural population of maize [6]. With the successful completion of the B73 genome sequencing project, more than 32,000 genes have been identified [7]; this provides a good opportunity for QTL cloning and function verification. However, the identification of genes related to grain yield is still a great challenge because of complexity of this trait. QTLs cloned to date suggest that genes with various functions can affect grain yield, including genes involved in protein degradation [8,9], hormone metabolism [10,11], and other processes [12,13]. So many genetic factors affect final grain yield that it has even been suggested that the entire genome may be involved [14].

Comparative QTL mapping studies have shown that some QTL for many traits, including grain yield, are located on collinear chromosomes in different species [15-18]. This suggests that mutations in orthologous genes contribute to similar trait variation. If the current, similar function of the genes was gained following divergence of the different species being compared, the mutations will be completely independent, and may be dissimilar in nature. This has been seen in the well-known "green revolution" genes *Rht *in wheat, *GAI *in *Arabidopsis *and *Dwarf8 *in maize, which all contribute to short plant stature [19].

The availability of the rice genomic sequences [20,21] facilitated the identification of many genes controlling grain size and weight [9,22-25]. The *GW2 *gene was the first gene controlling grain width to be cloned in rice. It encodes a RING-type protein with E3 ubiquitin ligase activity, and functions as a negative regulator of grain width and weight. A 1 bp deletion in the fourth exon resulting in a premature stop codon and a truncation of 310 amino acids causes the increase in rice grain width and weight [9]. Only one copy of *GW2 *exists in rice, but a search of the latest B73 genomic sequence identified two homologous gene sequences [7]. Whether both maize genes represent co-orthologs of the rice *GW2 *gene has not been tested. Besides, based on the conserved function found for various other genes, it is reasonable to suppose that the maize orthologs of rice *GW2 *may also affect grain size and weight, which needs to be tested further.

Association analysis can identify genetic polymorphisms that are associated with phenotypic variation. Compared with linkage analysis, it is time- and cost-effective. More importantly, it can investigate more than two alleles at the same time and can reach extraordinarily high resolution in species with rapid linkage disequilibrium (LD) decay [26]. Maize contains abundant genetic diversity and LD decays within 2 kb in diverse material [27-29]. This rapid LD decay pattern makes maize an ideal plant for association analysis to identify causal polymorphisms or closely linked polymorphisms, which can then be used to develop functional markers for marker-assisted selection [30]. To date, association analysis has been widely used in maize to dissect the genetic basis of complex traits, such as kernel carotenoid content [30], kernel starch content [31], kernel quality traits [32] and flowering time [33].

The objectives of this study were to clarify the relationship of the two maize genes that were found to be homologous to the rice *GW2 *gene; to investigate their evolutionary fate following the duplication of these genes in maize; and to characterize the contribution and putative function of these two genes in maize grain yield-related traits.

## Results

### The rice *GW2 *gene has two co-orthologs located on duplicated chromosomes in maize

Blast searches with the rice GW2 protein sequence [GenBank: ABO31101] against the maize high throughput genomic sequence database [34] identified two maize bacterial artificial chromosome (BAC) clones, AC212189 on chromosome 4 and AC211190 on chromosome 5, that contain sequences showing high similarity to the rice protein. The structures of these two genes were determined using three maize complementary DNA (cDNA) clones from GenBank, EU968771, FJ573211 and EU962093, which showed high sequence similarity to the two BAC clones. Both genes consist of eight exons, with an overall sequence similarity of 94% to each other and 93% to the rice *GW2 *gene across the coding region. They were named *ZmGW2-CHR4 *and *ZmGW2-CHR5*, based on their locations on the maize chromosomes (See additional file 1: Similarity between *ZmGW2-CHR4 *and *ZmGW2-CHR5 *across the cDNA region).

**Figure 1:**
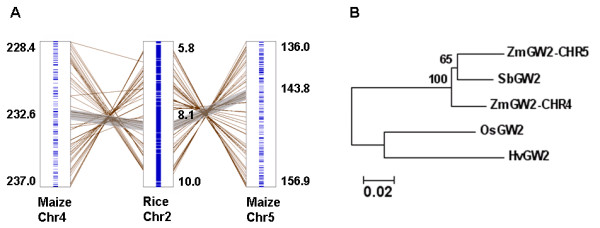
**Relationship of *ZmGW2-CHR4 *and *ZmGW2-CHR5***. A. Collinear relationship among genes in the surrounding regions of *GW2 *in maize and rice. Genes that show high similarity are connected by lines. The physical coordination of the sequences used to construct the collinear relationship and the coordination of *GW2 *are given along the chromosomes (units, Mb). The grey shades highlight the collinear regions near *GW2*. B. Phylogenetic tree of GW2 protein sequences in maize, rice, sorghum and barley. Numbers at the branches are percentages based on 1,000 bootstrap repetitions, bootstrap values >50% are given. The scale bar indicates the number of amino acid substitutions per position. Zm, *Zea mays*, Sb, *Sorghum bicolor *[GenBank: XM_002453553], Os, *Oryza sativa *[GenBank: EF447275], Hv, *Hordeum vulgare *[GenBank: EU333863].

Because the maize genome has been shown to be replete with duplicated chromosomal regions [7,35], we investigated if the two homologs represent duplicated genes. As shown in Figure 1A, other gene sequences in the vicinity of the two maize genes also show high similarity, as would arise following an ancient chromosome duplication event. Comparison of the regions around both maize genes with the region containing rice *GW2 *showed that both maize regions are collinear with the rice region, indicating that the two maize genes are co-orthologs of the rice *GW2 *gene. Besides, compared to the rice region containing *GW2*, both maize regions contain an inversion (Figure 1A).

Phylogenetic analysis of the duplicated maize genes and their corresponding counterparts in sorghum, rice and barley showed that *ZmGW2-CHR5 *is more closely related to the sorghum counterpart than to the other maize copy (Figure 1B), indicating that sequence divergence has occurred between the two maize genes. To identify the fixed polymorphic sites between the maize genes, we sequenced the coding regions of both genes in eight diverse maize lines belonging to five heterotic groups. In total, we found 70 fixed sites, of which 44 were synonymous mutations (Table 1). All polymorphisms but two were SNPs. The 70 sites are not evenly distributed across the entire coding region, as 60% of the polymorphisms occurred in exon 8, and no polymorphisms were found in exon 2 (Table 1). Only three SNPs were found in the RING domain, all of which were synonymous mutations, suggesting that this domain may have a conserved function in both genes.

**Table 1:** Distribution of sequence polymorphisms between *ZmGW2-CHR4 *and *ZmGW2-CHR5*

Exons	**E1-1**^**a**^	**E1-2**^**b**^	**E2**^**b**^	**E3**^**b**^	**E4-1**^**b**^	E4-2	E5	E6	E7	**E8**^**c**^	Total
Synonymous	6	1	0	2	0	2	6	2	0	25	44
Non-synonymous	4	0	0	0	0	2	0	1	2	17	26
Total	10	1	0	2	0	4	6	3	2	42	70

E, exon

^a ^Including a 6 bp InDel

^b ^The location of the RING domain, which begins from within the end of exon 1 (E1-2) and extends to within the beginning of exon 4 (E4-1)

^c ^Including a 3 bp InDel

### *ZmGW2-CHR4 *is located within a QTL for HKW

**Figure 2:**
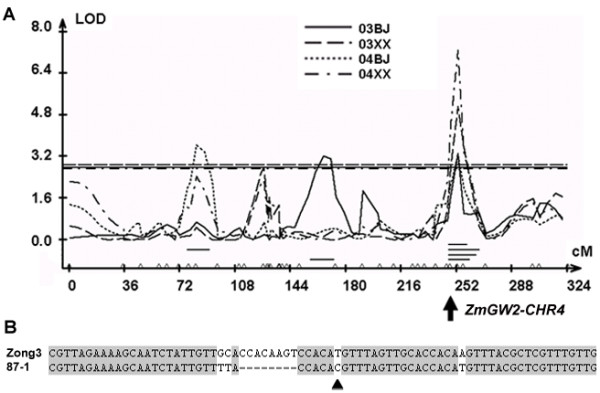
**QTL analysis in the IF_2 _population**. A. QTL profile for HKW generated from QTL Cartographer. 03, year 2003; 04, year 2004; BJ, Beijing; XX, Xunxian. B. Polymorphisms between the two parental lines Zong3 and 87-1 used to develop the IF_2 _population in the promoter region of *ZmGW2-CHR4*. The triangle represents site S40 (see details in Table 5).

*ZmGW2-CHR4 *and *ZmGW2-CHR5 *were located in maize chromosomal bins 4.09 and 5.04, respectively. Previous studies have mapped many QTL for kernel weight to these two regions (See additional file 2: QTL for grain yield mapped in previous studies in maize bins 4.09 and 5.04). In particular, in an F_2:3 _population [37] and an immortalized F_2 _(IF_2_) population developed by our lab at the China Agricultural University [40,41], a QTL for HKW was mapped to bin 4.09 (Figure 2A; Table 2). *ZmGW2-CHR4 *was mapped to 248 cM on chromosome 4, between simple sequence repeat (SSR) markers bnlg292 and umc1173, and within the QTL confidence interval for HKW, near the left border of the QTL (Figure 2A; Table 2). This QTL was identified stably across four seasons and explained 3.2% to 6.9% of the phenotypic variation in the IF_2 _population derived from inbred lines Zong3 and 87-1. The Zong3 allele can increase HKW from 0.5 to 1.3 g (Table 2). Although the large QTL confidence interval (10.4 ~ 18.7 cM) contained lots of genes, the involvement of *GW2 *in HKW in rice and the co-location of *ZmGW2-CHR4 *with this QTL indicated that *ZmGW2-CHR4 *is a good candidate for this QTL and may be involved in grain weight variation.

**Table 2:** QTL for HKW mapped in the IF_2 _population

Environments	Position (cM)	Confidence interval (cM)	LOD	**Additive**^**a**^	**Dominant**^**b**^	**R**^**2 **^**(%)**^**c**^
03BJ	252.5	248.0-260.4	3.3	0.7	0.5	3.3
03XX	252.5	248.0-264.3	5.1	1.2	1.1	5.1
04BJ	252.5	248.0-266.7	3.3	0.5	0.6	3.2
04XX	252.5	248.0-258.4	7.3	1.3	0.9	6.9

03, year 2003; 04, year 2004; BJ, Beijing; XX, Xunxian

^a ^Positive values indicate that the Zong3 allele increases HKW

^b ^Positive values indicate that the heterozygote has higher phenotypic value than the mean of the two homozygotes

^c ^Phenotypic variation explained by each QTL

### Genetic diversity and LD of the two genes across maize inbred lines

**Figure 3:**
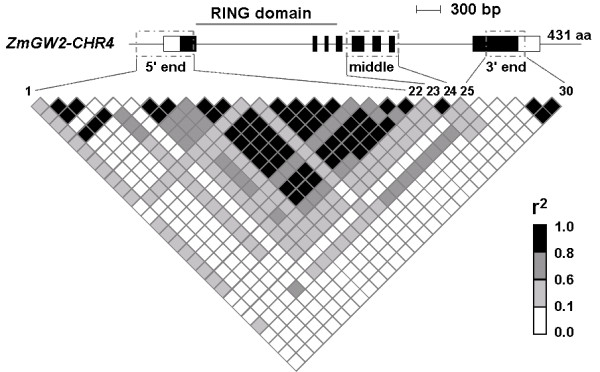
**Pattern of pairwise LD in *ZmGW2-CHR4***. All polymorphisms with minor allelic frequency exceeding 0.05 were used. In the gene diagram, filled black boxes represent exons, and open boxes indicate the untranslated regions (UTRs). Grey dash-dot boxes mark the regions sequenced in this study. Polymorphisms 1 to 22 are from the 5' end, 23 and 24 from the middle region, and 25 to 30 from the 3' end. The polymorphisms and their locations on the gene diagram are connected by lines.

**Figure 4:**
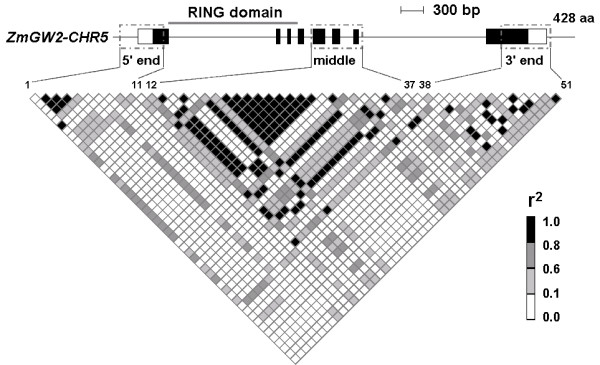
**Pattern of pairwise LD in *ZmGW2-CHR5***. All polymorphisms with minor allelic frequency exceeding 0.05 were used. In the gene diagram, filled black boxes represent exons, and open boxes indicate the UTRs. Grey dash-dot boxes mark the regions sequenced in this study. Polymorphisms 1 to 11 are from the 5' end, 12 to 37 from the middle region, and 38 to 51 from the 3' end. The polymorphisms and their locations on the gene diagram are connected by lines.

The genetic diversity within the RING domain was not analyzed because it is well conserved across the two maize genes (Table 1). Instead, we analyzed three regions of comparable sizes in corresponding regions of the two maize genes (Figure 3; Figure 4). The average levels of nucleotide diversity (π) in the two genes are comparable, both having about 4 nucleotide differences per 1,000 sites between two random sequences across the entire genes (Table 3). However, the nucleotide diversity is not evenly distributed across both genes. While the 5' end showed the most abundant diversity in *ZmGW2-CHR4 *(π = 7.7 × 10^-3^), this region showed the fewest polymorphisms in *ZmGW2-CHR5 *(π = 2.6 × 10^-3^). The middle portion of *ZmGW2-CHR4 *has only two polymorphisms, while the corresponding region of *ZmGW2-CHR5 *has 28 polymorphisms and the highest level of genetic diversity. Tajima's *D *statistic was calculated to determine whether the two genes were subjected to selective constraints. As shown in Table 3, it appears that neither gene has been the subject of natural selection when the entire gene sequences were analyzed. Because the selection effect may not extend throughout the entire gene [42], we calculated Tajima's *D *statistic separately for the three regions in each gene. The results showed that the middle portion of *ZmGW2-CHR4 *(Figure 3) has a significant positive Tajima's *D *value, suggesting the presence of selection at this region, which is consistent with the observed low level of nucleotide diversity (Table 3).

**Table 3:** Nucleotide diversity and neutrality test of *ZmGW2-CHR4 *and *ZmGW2-CHR5*

Gene	Region	**N**^**a**^	**Size (bp)**^**b**^	**S**^**c**^	**H**^**d**^	**π (× 10**^**-3**^**)**^**e**^	**Tajima's *D***^**f**^
*ZmGW2-CHR4*	5' end	116	689	22	11	7.7	0.80
	middle	118	665	2	2	1.5	2.51*
	3' end	115	495	5	6	1.8	-0.08
	Total	112	1849	29	21	3.9	0.97
*ZmGW2-CHR5*	5' end	111	585	9	9	2.6	-0.47
	middle	117	694	28	7	6.3	-0.51
	3' end	120	658	12	7	3.4	0.01
	Total	109	1937	46	19	4.2	-0.27

*, P < 0.05

^a ^The number of analyzed maize inbred lines

^b ^The total number of sites (excluding sites with gaps/missing data)

^c ^The number of segregating sites (excluding InDels)

^d ^The number of haplotypes

^e ^The number of nucleotide differences per site between two randomly chosen sequences

^f ^The *D *value calculated according to the polymorphic sites

The LD decay patterns of the two genes shown in Figures 3 and 4 indicate that both genes contain discrete LD blocks. In *ZmGW2-CHR4*, a large LD block was observed at the 5' end (Figure 3), and in *ZmGW2-CHR5*, a large LD block was observed in the middle portion (Figure 4). The only two polymorphisms in the middle portion of *ZmGW2-CHR4 *are in complete LD; thus, only two haplotypes were observed (Table 3; Figure 3), consistent with the observed selection and reduced nucleotide diversity in this region. Although LD extends in each of the three regions in both genes (less than 800 bp), LD among the three regions within both genes was within 2,000 bp (Figure 3; Figure 4), consistent with previous results [27-29]. We further investigated LD between the two genes, only 1.4% of r^2 ^values for all pairs of polymorphisms was greater than 0.10, and the largest r^2 ^was less than 0.16.

### Association analysis of four yield-related traits

Analysis of variance (ANOVA) showed significant phenotypic variation for all four yield-related traits among the maize lines studied (Table 4), indicating that the assembled panel is suitable for association analysis. A significant year by genotype effect was observed for all four traits. The phenotypic distributions ranged from 6.48 to 11.58 mm for KL, 5.65 to 10.57 mm for KW, 3.14 to 6.73 mm for KT and 7.87 to 42.86 g for HKW; with an average of 8.90 mm, 8.12 mm, 4.70 mm and 22.50 g, respectively (Table 4). Significant positive phenotypic and genetic correlations between KW and KT, and between kernel size traits (KL, KW and KT) and HKW were observed (Table 4), indicating that an increase in any of the three kernel size traits can increase HKW, and thus perhaps grain yield.

**Table 4:** Mean squares of ANOVA and descriptive statistics and correlation coefficients for four yield-related traits

Category	Source of variation	DF	KL (mm)	KW (mm)	KT (mm)	HKW (g)
ANOVA	Year	2	52.09**	0.45**	33.78**	755.90**
	Genotype	120	1.84**	2.40**	0.87**	72.02**
	Replication (Year)	3	0.08	0.18	0.13	84.25**
	Year × Genotype	222 (215)^a^	0.41**	0.23**	0.18**	16.59**
	Error	287 (298)^a^	0.11	0.09	0.07	6.45
Descriptive	Range		6.48-11.58	5.65-10.57	3.14-6.73	7.87-42.86
statistics	Mean ± SD		8.90 ± 0.84	8.12 ± 0.76	4.70 ± 0.60	22.50 ± 4.99
Correlation	KL (mm)			0.16	-0.15	0.37**
coefficients^b^	KW (mm)		0.17**		0.51**	0.78**
	KT (mm)		-0.09*	0.36**		0.62**
	HKW (g)		0.26**	0.68**	0.58**	

DF, degree of freedom; HKW, one-hundred kernel weight; KL, kernel length; KT, kernel thickness; KW, kernel width; **, P < 0.01; *, P < 0.05

^a ^Because the phenotypic values for some lines were missing either for kernel size or for kernel weight, the DF are different. The numbers in parentheses indicate the DF for HKW and the numbers outside parentheses indicate the DF for KL, KT and KW

^b ^The numbers above the diagonal are genetic correlation coefficients and the numbers below the diagonal are phenotypic correlation coefficients

The mixed model controlling for population structure (Q) and kinship (K) as estimated using molecular markers [43] was employed to test associations between the four yield-related traits and polymorphisms from the two maize genes. Out of 360 (30 sites × 4 traits × 3 years) and 612 possible associations (51 sites × 4 traits × 3 years), 81 and 49 associations were significant for *ZmGW2-CHR4 *and *ZmGW2-CHR5*, respectively, at P ≤ 0.05; while at P ≤ 0.01, 33 and 22 associations remained significant, indicating that the observed associations were not expected only by chance.

Taking the LD (r^2 ^> 0.8) level among sites into account, seven and five sites from *ZmGW2-CHR4 *and *ZmGW2-CHR5*, respectively, were significantly associated with at least one of the four yield-related traits at P ≤ 0.01. Information on the location, genotype, frequency and probability value for each site can be found in Tables 5 and 6. The S40 site from *ZmGW2-CHR4 *was of great interest because it showed associations with KW and HKW across all three field experiments (Table 5), and explained 8.0% and 11.4% of the phenotypic variation for KW and HKW, respectively in Beijing in 2007. This site also segregated in the IF_2 _population and mapped to a region where a QTL for HKW was identified (Figure 2B). Three other sites from *ZmGW2-CHR4 *(S27, S304 and S1730) and one site from *ZmGW2-CHR5 *(S1789) were significantly associated with KW in two out of the three field experiments; and sites S628 from *ZmGW2-CHR4 *and S1632 from *ZmGW2-CHR5 *showed significant effects on KT in two out of the three field experiments. Other trait/site associations identified in this study were significant in only one field experiment and could not be repeated across years and locations (Table 5; Table 6).

**Table 5:** Associations between yield-related traits and the polymorphisms of *ZmGW2-CHR4*

Site	Location	**Genotype**^**a**^	Frequency	Env	**KW**^**b**^	**KL**^**b**^	**KT**^**b**^	**HKW**^**b**^	**Zong3/87-1**^**c**^
S27	Promoter	0/**8**	22/95	07BJ	1.07E-04			0.0029	0/**8**
				07HN					
				08HN	0.0090				
S40	Promoter	**C**/T	18/99	07BJ	3.32E-04	0.0353		2.24E-04	T/**C**
				07HN	0.0137		0.0226	0.0498	
				08HN	0.0086		0.0054	0.0242	
S304	Promoter	**C**/T	19/97	07BJ	0.0021	0.0058		0.0037	T/**C**
				07HN					
				08HN	0.0196				
S338	Promoter	**0**/1	26/90	07BJ				0.0352	1/**0**
				07HN			0.0088		
				08HN					
S628	Exon 1	C/**T**	95/21	07BJ				0.0127	C/C
				07HN	0.0356		0.0102		
				08HN			0.0078		
S1730	Exon 8	C/**T**	95/20	07BJ	0.0066	0.0446		4.22E-04	C/C
				07HN	0.0087		0.0180		
				08HN					
S1865	3' UTR	0/**1**	95/20	07BJ		0.0050	0.0292		0/0
				07HN	0.0198			0.0075	
				08HN					

Env, environment; HKW, one-hundred kernel weight; KL, kernel length; KT, kernel thickness; KW, kernel width; 07BJ, year 2007 Beijing; 07HN, year 2007 Hainan; 08HN, year 2008 Hainan

^a ^The numbers indicate the number of deleted nucleotides, and the letters indicate nucleotides. Numbers and letters in bold indicate the favourable alleles with the exception of the S1865/07BJ-KL association

^b ^Significant probabilities

^c ^The allele of inbred lines Zong3 and 87-1 which were used to develop an IF2 population for QTL mapping [40,41]. Letters and numbers in bold indicate the favourable alleles

**Table 6:** Associations between yield-related traits and the polymorphisms of *ZmGW2-CHR5*

Site	Location	**Genotype**^**a**^	Frequency	Env	**KW**^**b**^	**KL**^**b**^	**KT**^**b**^	**HKW**^**b**^	**Zong3/87-1**^**c**^
S908	Intron 5	0/**1**	101/16	07BJ					1/0
				07HN					
				08HN	0.0054	0.0024			
S1601	Exon 8	**A**/T	22/98	07BJ					A/T
				07HN					
				08HN	0.0055	0.0018			
S1632	Exon 8	**A**/C	14/106	07BJ			0.0464		C/C
				07HN			0.0087		
				08HN					
S1789	3' UTR	0/**17**	105/15	07BJ	0.0394				17/0
				07HN					
				08HN	0.0032	0.0043			
S2051	3' flanking	**0**/13	27/93	07BJ					0/0
				07HN					
				08HN	0.0067			0.0363	

Env, environment; HKW, one-hundred kernel weight; KL, kernel length; KT, kernel thickness; KW, kernel width; 07BJ, year 2007 Beijing; 07HN, year 2007 Hainan; 08HN, year 2008 Hainan

^a ^The numbers indicate the number of deleted nucleotides, and the letters indicate nucleotides. Numbers and letters in bold indicate the favourable alleles

^b ^Significant probabilities

^c ^The allele of inbred lines Zong3 and 87-1 which were used to develop an IF2 population for QTL mapping [40,41]

We further investigated whether the directions of effects in the association population were the same as what were predicted by the QTL mapping analysis of the IF_2 _population (Figure 2; Table 2). Of the seven significant polymorphisms associated with HKW from *ZmGW2-CHR4*, four segregated in the IF_2 _population and could be tested. In the association panel, all four favourable alleles are from the inbred line 87-1 (Table 5), while in the IF_2 _population, the allele that increases HKW is from the other parent, Zong3 (Table 2). For *ZmGW2-CHR5*, we did not detect any QTL for HKW in its vicinity in the IF_2 _population. Consistent with this result, the only polymorphism that showed significant association with HKW in the association panel did not segregate in the IF_2 _population (Table 6).

### Expression analyses of the two maize genes

**Figure 5:**
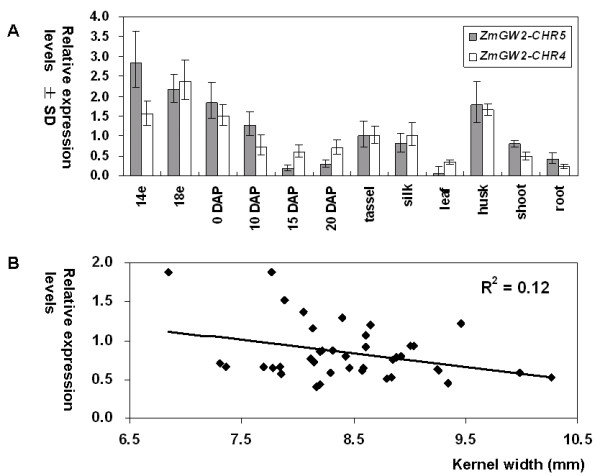
**Expression analysis of *ZmGW2-CHR4 *and *ZmGW2-CHR5***. A. Tissue expression patterns. 14e, 18e, ears from plants with 14 and 18 expanded leaves, respectively. DAP, days after pollination. The height of each column represents the relative expression level of the corresponding gene. B. Correlation between the expression levels of *ZmGW2-CHR4 *and kernel width. Kernels collected at 0 DAP were used for expression analysis.

Real-time quantitative reverse transcription PCR (qRT-PCR) of 12 different tissues was performed to address whether the two maize genes had diverged expression patterns and whether these patterns were associated with a role in kernel development. As shown in Figure 5A, expression trends of the two genes were quite similar, with a correlation coefficient of 0.84, indicating no divergence between the two genes in the tissues examined. The highest expression levels were observed in immature ears for both genes, and expression levels were reduced in kernels after pollination, suggesting a role in kernel development. Correlation analysis between the expression levels of the two genes and the four traits across 41 maize inbred lines showed that *ZmGW2-CHR4 *transcript abundance was negatively correlated with KW (Figure 5B, N = 41, P = 0.03, R^2 ^= 0.12). However, despite the correlation between KW and the expression level of *ZmGW2-CHR4*, and the association between KW and six *ZmGW2-CHR4 *polymorphisms (Table 5), none of these polymorphisms affected the expression level of *ZmGW2-CHR4*. No significant correlations were observed between *ZmGW2-CHR5 *transcript levels and any of the four traits.

## Discussion

### Evolution of the two maize genes

The collinear relationship between the rice region containing *GW2 *and the maize regions containing *ZmGW2-CHR4 *and *ZmGW2-CHR5 *suggested that the two maize genes are duplicated genes and both of them are co-orthologs of rice *GW2 *(Figure 1A). Previous studies consistently showed that maize has a segmental allotetraploid origin, in which the maize genome was thought to have arisen through hybridization of two ancestral diploids whose genomes had partially diverged, one of which shares a more recent common ancestor with the sorghum genome [44,45]. To clarify the relationship of the *GW2 *genes in maize and sorghum, we performed phylogenetic analysis with the GW2 protein sequences, which showed that *ZmGW2-CHR5 *is more closely related to its counterpart in sorghum than to *ZmGW2-CHR4 *(Figure 1B). This finding supports the segmental allotetraploid origin of maize, and indicates that the two maize genes may have evolved independently for a period of time [44,45].

Three processes have been proposed to explain the evolutionary fates of duplicated genes within a species: non-functionalization, neo-functionalization and sub-functionalization. In non-functionalization, one of the duplicates accumulates deleterious mutations and eventually degenerates to a pseudogene or is lost from the genome [46-48]. Occasionally, mutations in regulatory or coding regions can lead to novel gene function (neo-functionalization) [48,49]. Alternatively, both genes may experience some degeneration and lose partial functionality, but can complement each other (sub-functionalization). This can occur through partition of either protein domains or regulatory elements of the ancestral gene [50-52]. Sequence analyses showed that *ZmGW2-CHR4 *and *ZmGW2-CHR5 *are highly conserved, with an overall similarity of 94% across the coding region. None of the mutations in either gene led to truncated proteins, and no non-synonymous nucleotide changes were observed in the RING domain. Additionally, both genes are expressed across various tissues. Thus, neither of the genes has experienced a non-functionalization process.

To assess if neo-functionalization and sub-functionalization have occurred, previous knowledge of the ancestral gene's function is required, which is usually unavailable. Besides, both processes can occur through changes in regulatory or coding regions. Therefore, we tested and distinguished the two processes by investigating whether reciprocal degenerations have occurred. For example, if one gene was expressed in roots and silenced in leaves, sub-functionalization requires that the other gene must be silenced in roots and expressed in leaves (occurrence of reciprocal degeneration); while neo-functionalization requires that the other gene must be silenced in both tissues so that the expression in roots could be assumed to be a new function. In *ZmGW2-CHR4 *and *ZmGW2-CHR5*, the exon number, protein length and protein domain are all well conserved (Table1; Figure 3; Figure 4), with no protein domains lost or acquired, indicating that neo-functionalization or sub-functionalization through changes in the coding region had not occurred. We further investigated the expression patterns of the two genes across 12 maize tissues (Figure 5A). Although the promoter regions of the two genes are highly divergent, both genes were expressed in all of the 12 tissues and their expression levels were highly correlated (r = 0.84), indicating that neither neo-functionalization nor sub-functionalization through changes in regulatory regions had occurred in the tissues examined. However, we could not exclude the possibility that the two genes may show divergent expression patterns under various environmental conditions or in specific cell types which have not been tested.

### Genetic variations in *ZmGW2-CHR4 *and *ZmGW2-CHR5 *are associated with kernel size and weight in maize

The orthologous relationship and high sequence conservation between the two maize genes and the rice *GW2 *suggest that *ZmGW2-CHR4 *and *ZmGW2-CHR5 *may perform similar functions as *GW2 *in rice. Results from expression, linkage and association analyses corroborate this supposition. The expression levels of both maize genes varied according to the developmental stages of the ear or kernel (Figure 5A), implying that they may be involved in ear or kernel development. This was further supported by association analysis. Although the small size of the association mapping panel did not guarantee optimal power of association tests [53], both genes were found to contain polymorphisms affecting at least one of the four yield-related traits (Table 5; Table 6). Two more lines of evidence were presented to support a role of *ZmGW2-CHR4 *in kernel size and weight. One came from QTL mapping results (Figure 2; Table 2), which placed *ZmGW2-CHR4 *within the confidence interval of a consistent QTL for HKW. Another came from the negative correlation between the expression levels of *ZmGW2-CHR4 *and KW (Figure 5B), indicating that down-regulation of this gene may lead to elevated KW, and thus grain yield. This is consistent with the negative function of *GW2 *in rice, where a truncated protein leads to an increase in grain size and weight [9].

Previous study with another yield-related gene, *GS3*, showed that different polymorphisms underlie similar phenotypes in rice and maize [6]. Here, we report a similar phenomenon. In rice, a 1 bp deletion in the fourth exon leads to a premature truncated protein and causes enhanced grain width and weight [9], while in maize, mutations in other regions of *ZmGW2-CHR4 *and *ZmGW2-CHR5 *cause those phenotypes (Table 5; Table 6). However, we cannot exclude the possibility that the 1 bp deletion found in the rice *GW2 *gene also exists in maize, and would have been found if more (and more diverse) lines are sequenced for these genes. Moreover, the genetic polymorphisms that were associated with phenotypic variation for kernel size and weight are different between the two maize genes, indicating that they probably affect kernel size and weight through different mechanisms.

In QTL analysis, the favourable allele for HKW is from the inbred line Zong3 (Table 2). In our association panel, the favourable alleles of all four polymorphisms that segregated in the QTL mapping population were from the other parental inbred line, 87-1 (Table 5). Three possible explanations may explain the discrepancy in the direction of allelic effects. One is that the associations are false positives created due to the possible presence of population structure and individual relatedness. The best statistical model to account for the effect of population structure and individual relatedness and control the false positive rate is the mixed linear model [43,53]. This was the model used in this study, with which we found that one polymorphism was consistently associated with HKW across three different environments, and the direction of the allelic effect was the same across these environments (Table 5). For complex traits, such as HKW, the consistent detection of a significant association across various environments using a well-performed statistical model implies that the associations are not false positives.

A second possibility for the discrepancy is that we may not have identified the actual functional polymorphism, but rather only one linked to it. Our evidence shows that *ZmGW2-CHR4 *and *ZmGW2-CHR5 *are associated with kernel size and weight in maize. However, it is still unclear what the actual causal polymorphism(s) is (are) in each case because association analysis based on LD can identify neutral polymorphisms (hereafter referred to as "the detected polymorphism") in high LD with the actual functional polymorphism (See additional file 3: LD between sites significantly associated with kernel size and weight in *ZmGW2-CHR4*; additional file 4: LD between sites significantly associated with kernel size and weight in *ZmGW2-CHR5*). When the detected polymorphism is in complete LD with the functional polymorphism, the favourable allele at the detected polymorphism can represent the favourable allele at the functional polymorphism completely. However, if recombination occurred between the two polymorphisms, some inbred lines would show a favourable allele at the detected polymorphism, but actually contain the unfavourable allele at the functional polymorphism. This cannot be detected in association analysis where the mean effects of all lines are measured, but can be detected in linkage analysis where only two lines (this is, lines where recombination occurred between the functional polymorphism and the detected polymorphism) are involved.

A third possibility is the presence of other functional polymorphisms that we did not detect in the present study. The interval of a QTL usually spans 10-30 cM [54], and can contain more than one gene involved in the expression of the same trait in some cases [10,55]. Thus, the QTL effect actually represents the combined effect of all functional polymorphisms within the region. In the association analysis that included Zong3 and 87-1, we detected favourable alleles in *ZmGW2-CHR4 *from 87-1, but if there were other unfavourable functional polymorphisms in *ZmGW2-CHR4 *or other possible genes within the QTL that were not measured in the association analysis, the mean effect would identify 87-1 as the unfavourable parent in the QTL mapping population. This is quite possible, because our correlation analysis also pointed to the presence of other functional polymorphisms. In this study, we found a significant negative correlation between the expression levels of *ZmGW2-CHR4 *and KW, but this correlation cannot be explained by the identified significant polymorphisms in the association analysis, implying the presence of other causal polymorphisms. These polymorphisms could be *cis*-acting elements far upstream of the genes, as in the case of *tb1 *[56]. Alternatively, they could be unknown *trans*-acting elements hidden in the genome, as have been reported in the maize genome [57]. However, it is very difficult to identify upstream *cis*- or *trans*-acting elements using the candidate gene association analysis strategy. An alternative method to identify these elements and to further explore the genetic basis of complex quantitative traits is genome-wide association studies with high density marker coverage [58].

## Conclusions

This study investigated the relationship, evolutionary fate and function of two maize genes involved in kernel size and weight. The two genes represent chromosomal duplicates that are co-orthologs of *GW2 *in rice. The sequences of both genes are well conserved, with no mutations leading to a pseudo-molecule, and no new protein motifs were found, suggesting that both genes may have conserved functions in maize. Expression and candidate gene-based association analyses suggested that both genes play a role in kernel size and weight variation, as does rice *GW2*. However, the identified polymorphisms that contribute to phenotypic variation are different between the maize and rice genes and between the two maize genes, suggesting that the three genes may cause phenotypic variation through different mechanisms. Mutant or transformation experiments would shed more light on this hypothesis. The conservation of function (all associated with variation in kernel size and weight) together with the diversification of mechanism (different identified polymorphisms) among the three genes can help us to understand the similarities as well as the differences in the genetic basis of grain yield in rice and maize.

## Methods

### Determining the relationship between the two maize genes

Sequences in the vicinity of the two maize genes and the rice *GW2 *gene were used to generate the collinear relationships among them. Because the three regions have different degrees of chromosomal expansion, different lengths of sequences were used. Specifically, a stretch of 8.6 Mb on maize chromosome 4 (B73 genome, 228.4-237.0 Mb), 20.9 Mb on maize chromosome 5 (B73 genome, 136.0-156.9 Mb) and 4.2 Mb on rice chromosome 2 (*Oryza sativa japonica *TIGR5, 5.8-10.0 Mb) were used. The online program SyMAP v3.0 [59] was used to draw and display the collinear relationship among the three regions with default settings.

The homologous sequences of the rice *GW2 *gene from other species, including maize, sorghum and barley, were obtained via BLAST analysis in NCBI [34] and PlantGDB [60]. The phylogenetic tree was generated using MEGA, version 3.1 [61] with the neighbor-joining method, Kimura two-parameter distance and pairwise deletion analysis. Robustness of the constructed phylogenetic tree was tested with 1,000 bootstrap repetitions of the informative polymorphisms.

### Genetic mapping and QTL analysis

A primer pair, M9 (See additional file 5: Primers used in this study) was designed from the sequences of *ZmGW2-CHR4 *and used to map the gene in the recombinant inbred line (RIL) population derived from Zong3 and 87-1 [62] using MAPMAKER/EXP 3.0 [63]. The RIL population was used to develop an IF_2 _population consisting of 441 crosses to map QTL and heterotic loci for yield-related traits [40,41]. The IF_2 _genotypes were deduced according to the marker genotypes of their RIL parents, and the detailed method and the IF_2 _design can be found in a previous study [41]. The composite interval mapping model [64] implemented in QTL Cartographer 2.5 [65] was used to map QTL for HKW in the IF_2 _population following addition of the M9 marker. A window size of 10 cM, 5 control markers and the forward regression method were used. The threshold for declaring a QTL was determined by 1,000 random permutations with a significance level of 0.05.

### Sequencing and analyses

Coding regions of the two genes in eight maize inbred lines were sequenced in order to identify the fixed sites, which were the sites showing polymorphisms between the two maize genes but showing no variation within each gene across a panel of diverse lines. These lines were from the five main heterotic groups in China [66], including three lines from the TangSPT group (K12, Hai014 and S22), two from the Reid group (Shen5003 and 812) and one each from the Lancaster (4F1), Temp-tropic (SW1611) and Zi330 groups (5311). The heterotic groups were classified according to the genetic distance calculated using SSR markers. The genetic distance between lines within each group is closer than between lines from different groups. For the nucleotide diversity and LD analyses, three segments of each gene were sequenced, corresponding to the 5' end, the middle portion and the 3' end, across 121 lines with good agronomic performance among the assembled association mapping panel consisting of 155 diverse lines [53]. The primer pairs were designed in corresponding regions of the two homologous genes so that comparisons between them could be performed. Detailed information on the primers can be found in Additional file 5: Primers used in this study. Direct PCR products from the inbred lines in this study, which are almost completely homozygous across the whole genome, were sequenced. For ambiguous chromatograms, the products were sequenced again in the reverse direction, or the DNA was re-amplified and sequencing was repeated.

After sequencing, an initial alignment was performed with the multiple sequence alignment program MUSCLE [67] to detect singletons, which are polymorphisms that are found only once among the sequenced materials. Lines in which singletons were found were analyzed again until they were confirmed as correct. MUSCLE was then used again to align the confirmed sequences, which were subsequently refined manually in BioEdit [68]. Three parameters implemented in the DnaSP, version 4.00 [69] were used to measure genetic diversity: the average pairwise nucleotide difference per site - π, the number of segregating sites - S; and the number of haplotypes - h. Tajima's *D *statistic [70] was also calculated to investigate evidence for past selection. The LD level between sites with allelic frequency > 0.05 was calculated using TASSEL 2.0.1 [71].

### Field design and statistical analyses

A maize association mapping panel with 155 diverse inbred lines developed by Yang et al. [53] was used to find associations between the DNA polymorphisms in the two maize genes and grain yield components. Population structure (Q) and relative kinship (K) were reported in a previous study [53]. Briefly, population structure was inferred using 82 SSR markers in STRUCTURE 2.2 [72,73] with five independent runs at each k (number of populations, which was set from 1 to 10). Results indicated the presence of two sub-populations [53], which were incorporated into the Q matrix. The kinship matrix K was calculated using 884 SNP markers in SPAGeDi [74], and negative values between individuals were set to 0 [43]. Of the 155 inbred lines, 121 lines with good agronomic performance were phenotyped in the present study. Two of the field experiments, Beijing and Hainan in 2007, have been reported previously [6]. An additional field experiment with two replications in Hainan was performed in 2008 using similar field design and management as in Li et al. [6]. KL, KW, KT and HKW were measured as described by Li et al. [6]. The analysis of variance, descriptive statistics and correlation analysis for the four yield-related traits were performed using the SAS system (version 8.02, SAS Institute Inc., Cary, NC, USA). Association analysis was performed with TASSEL 2.0.1 [71] using the mixed model, Q+K [43].

### RNA extraction and qRT-PCR

Plant materials used to perform expression pattern analysis were described in a previous study [6]. Briefly, twelve tissues were collected from the inbred line 87-1, including seedling shoot, seedling root, mature leaf, tassel from plants with 15 expanded leaves, silk and husk from ears 0 days after pollination (DAP), immature ears from plants with 14 and 18 expanded leaves, and kernels harvested at four time points after pollination (0 DAP, 10 DAP, 15 DAP and 20 DAP). In addition, kernels at 0 DAP were collected from 41 inbred lines (See additional file 6: Materials used for correlation analysis) which were grown in a field in Shangzhuang, Beijing in 2007 to perform correlation analysis between expression levels and yield-related traits. TRIzol (Invitrogen, Carlsbad, California, USA) and RNase-free DNase (Promega, Madison, Wisconsin, USA) were used to prepare the total RNA, which was then used to synthesize the cDNA with the MMLV retro-transcriptase and an oligo (dT) primer (Promega). qRT-PCR was performed with the Ex Taq premix (Takara Shuzo, Kyoto, Japan) and primers listed in Additional file 5: Primers used in this study. These specific primers were designed based on sequence differences between the two maize genes, so that each primer pair will amplify only one of the genes. All experiments were carried out following the manufacturers' instructions. The 2^-ΔΔC^_T _method [75] was employed to calculate relative expression levels with the housekeeping gene ubiquitin as an endogenous control. Tassel and 0 DAP kernels from the inbred line 87-1 were used as the reference tissues in the expression pattern and correlation analyses, respectively. Three replicates were performed to calculate the average and standard deviation of expression levels for each sample.

## Abbreviations

ANOVA: analysis of variance; BAC: bacterial artificial chromosome; cDNA: complementary DNA; DAP: days after pollination; HKW: one-hundred kernel weight; IF_2: _immortalized F_2_; InDels: insertion/deletion polymorphisms; KL: kernel length; KT: kernel thickness; KW: kernel width; LD: linkage disequilibrium; qRT-PCR: real time quantitative reverse transcription PCR; QTL: quantitative trait locus; RIL: recombinant inbred line; SNP: single nucleotide polymorphism; SSR: simple sequence repeat; UTRs: untranslated regions.

## Authors' contributions

QL carried out the sequence, linkage, expression and association analyses and wrote the manuscript. LL performed the relationship analysis of the genes among species. GHB participated in field experiments and trait evaluation. XHY participated in association analysis. LL and XHY helped to prepare the materials. MLW gave critical suggestions to the interpretation of the results and helped to revise the manuscript. JBY designed the study. JRD and JSL participated in its design and coordination. JBY and JSL helped to draft the manuscript. All authors read and approved the final manuscript.

## Supplementary Material

Additional file 1 **Similarity between *ZmGW2-CHR4 *and *ZmGW2-CHR5 *across the cDNA region**. This is a figure showing the similarity between *ZmGW2-CHR4 *and *ZmGW2-CHR5 *across the cDNA region. The coding region is depicted using a filled grey box. The similarity was averaged over every 10 aligned nucleotides. From this figure, we can see that the coding region is well-conserved, while the 5' UTR and 3' UTR diverge between the two genes. Click here for file

Additional file 2 **QTL for grain yield mapped in previous studies in maize bins 4.09 and 5.04**. This is a table showing the QTL for grain yield mapped in previous studies in maize bins 4.09 and 5.04. Click here for file

Additional file 3 **LD between sites significantly associated with kernel size and weight in *ZmGW2-CHR4***. This is a table. It shows the LD level between sites significantly associated with kernel size and weight in *ZmGW2-CHR4*. Click here for file

Additional file 4 **LD between sites significantly associated with kernel size and weight in *ZmGW2-CHR5***. This is a table. It shows the LD level between sites significantly associated with kernel size and weight in *ZmGW2-CHR5*. Click here for file

Additional file 5 **Primers used in this study**. This is a table. It shows the primers used in this study. Click here for file

Additional file 6 **Materials used for correlation analysis**. This is a table. It shows the lines used for correlation analysis between the expression levels of *ZmGW2-CHR4 *and *ZmGW2-CHR5 *and the four yield-related traits. Click here for file
