# Supplementary figures and images for: Relationship, evolutionary fate and function of two maize co-orthologs of rice GW2 associated with kernel size and weight
Source: BMC Plant Biol. 2010 Jul 14;10:143. doi: 10.1186/1471-2229-10-143 (PMC3017803; doi:10.1186/1471-2229-10-143)

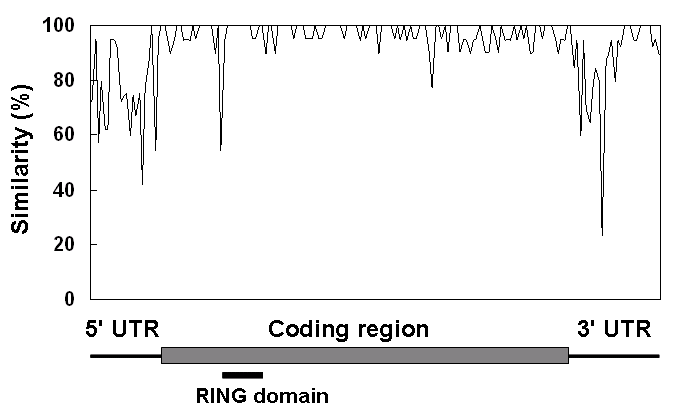

Supplement: Additional file 1 — Similarity between ZmGW2-CHR4 and ZmGW2-CHR5 across the cDNA region. This is a figure showing the similarity between ZmGW2-CHR4 and ZmGW2-CHR5 across the cDNA region. The coding region is depicted using a filled grey box. The similarity was averaged over every 10 aligned nucleotides. From this figure, we can see that the coding region is well-conserved, while the 5' UTR and 3' UTR diverge between the two genes. [file 1471-2229-10-143-S1.TIFF]
