# Supplementary material for: Relationship, evolutionary fate and function of two maize co-orthologs of rice GW2 associated with kernel size and weight
Source: BMC Plant Biol. 2010 Jul 14;10:143. doi: 10.1186/1471-2229-10-143 (PMC3017803; doi:10.1186/1471-2229-10-143)
Supplement: Additional file 2 — QTL for grain yield mapped in previous studies in maize bins 4.09 and 5.04. This is a table showing the QTL for grain yield mapped in previous studies in maize bins 4.09 and 5.04. [file 1471-2229-10-143-S2.DOC]

### Additional file 2 – QTL for grain yield mapped in previous studies in maize bins 4.09 and 5.04

| Bins | MaizeGDB ID | Trait | References |
| --- | --- | --- | --- |
| 4.09 | 854806 | kernel weight | [36] |
| 4.09 | ─ | kernel weight | [37] |
| 5.04 | 854808 | kernel weight | [36] |
| 5.04 | 86070 | grain weight | [38] |
| 5.04 | 86093 | grain weight | [38] |
| 5.04 | 86108 | grain weight | [38] |
| 5.04 | 86130 | grain weight | [38] |
| 5.04 | 86150 | grain weight | [38] |
| 5.04 | 850338 | dry matter yield | [39] |
