# Supplementary material for: Relationship, evolutionary fate and function of two maize co-orthologs of rice GW2 associated with kernel size and weight
Source: BMC Plant Biol. 2010 Jul 14;10:143. doi: 10.1186/1471-2229-10-143 (PMC3017803; doi:10.1186/1471-2229-10-143)
Supplement: Additional file 3 — LD between sites significantly associated with kernel size and weight in ZmGW2-CHR4. This is a table. It shows the LD level between sites significantly associated with kernel size and weight in ZmGW2-CHR4. [file 1471-2229-10-143-S3.DOC]

### *Additional file 3 – LD between sites significantly associated with kernel size and weight in* ZmGW2-CHR4

|  | S27 | S40 | S304 | S338 | S628 | S1730 | S1865 |
| --- | --- | --- | --- | --- | --- | --- | --- |
| S27 | 1 |  |  |  |  |  |  |
| S40 | 0.04 | 1 |  |  |  |  |  |
| S304 | 0.05 | 0.76 | 1 |  |  |  |  |
| S338 | 0.04 | 0.51 | 0.68 | 1 |  |  |  |
| S628 | 0.03 | 0.57 | 0.41 | 0.67 | 1 |  |  |
| S1730 | 0.05 | 0.66 | 0.47 | 0.53 | 0.71 | 1 |  |
| S1865 | 0.01 | 0.04 | 0.04 | 0.06 | 0.05 | 0.04 | 1 |
