# Supplementary material for: Relationship, evolutionary fate and function of two maize co-orthologs of rice GW2 associated with kernel size and weight
Source: BMC Plant Biol. 2010 Jul 14;10:143. doi: 10.1186/1471-2229-10-143 (PMC3017803; doi:10.1186/1471-2229-10-143)
Supplement: Additional file 4 — LD between sites significantly associated with kernel size and weight in ZmGW2-CHR5. This is a table. It shows the LD level between sites significantly associated with kernel size and weight in ZmGW2-CHR5. [file 1471-2229-10-143-S4.DOC]

### *Additional file 4 – LD between sites significantly associated with kernel size and weight in ZmGW2-CHR5*

|  | S908 | S1601 | S1632 | S1789 | S2051 |
| --- | --- | --- | --- | --- | --- |
| S908 | 1 |  |  |  |  |
| S1601 | 0.62 | 1 |  |  |  |
| S1632 | 0.01 | 0.03 | 1 |  |  |
| S1789 | 0.79 | 0.64 | 0.02 | 1 |  |
| S2051 | 0.39 | 0.27 | 0.04 | 0.49 | 1 |
