# Supplementary material for: Relationship, evolutionary fate and function of two maize co-orthologs of rice GW2 associated with kernel size and weight
Source: BMC Plant Biol. 2010 Jul 14;10:143. doi: 10.1186/1471-2229-10-143 (PMC3017803; doi:10.1186/1471-2229-10-143)
Supplement: Additional file 5 — Primers used in this study. This is a table. It shows the primers used in this study. [file 1471-2229-10-143-S5.DOC]

### Additional file 5 – Primers used in this study

| Category | Name | Sequences |
| --- | --- | --- |
| qRT-PCR | CHR5 | 5'-AGAGAGGAGGGAGAGTGCTCAA-3'/5'-TGCGCTTGCGTCCTACCA-3' |
|  | CHR4 | 5'-CCAGGTGGATATTCGTGTGCAG-3'/5'-GCACTCTCCCTCCTCTCTTACCA-3' |
|  | Ubiquitin | 5'-GGAAAAACCATAACCCTGGA-3'/5'-ATATGGAGAGAGGGCACCAG-3' |
| Full length cDNA amplification | GW2C | 5'-GAGCAAGGGTTTCGTCTCC-3'/5'-CAAGCCATGAAGCAACACAT-3' |
| *ZmGW2-CHR4* 5' end | 10F/3R | 5'-CGCTCAGCTGATTTTTGTGA-3'/5'-CACTCCTCAAGGTCGAGGTC-3' |
| *ZmGW2-CHR4* middle | M27 | 5'-TGCATGTCCAGCTTCTATGC-3'/5'-AATGCTGCATGTTGAAACCA-3' |
| *ZmGW2-CHR4* 3' end | M26 | 5'-GCGATTGCATGAGCTAACC-3'/5'-TACAGGACCCAAGAACACG-3' |
| *ZmGW2-CHR5* 5' end | M23 | 5'-AGAGGCTCCACAGTCGTTGT-3'/5'-GCTTACCAGGAAGCAGATGG-3' |
| *ZmGW2-CHR5* middle | M29 | 5'-CTGCAATCTGCATTTGCTGT-3'/5'-TTTAACTACAAATCCTGCACAC-3' |
| *ZmGW2-CHR5* 3' end | M24 | 5'-AGAAATGCCTCATCCAGGTG-3'/5'-AATGCCTGTTCCAGGTCAAC-3' |
|  | M25 | 5'-ATGGCTGGCAGTAGCACAT-3'/5'-CCGGTCCAGAGTCTAGTTGTT-3' |
| Mapping of *ZmGW2-CHR4* | M9 | 5'-AATATAATACCGAGAGGGCG-3'/5'-CACCTGGATGAGGCATTTCT-3' |
