# Supplementary material for: Relationship, evolutionary fate and function of two maize co-orthologs of rice GW2 associated with kernel size and weight
Source: BMC Plant Biol. 2010 Jul 14;10:143. doi: 10.1186/1471-2229-10-143 (PMC3017803; doi:10.1186/1471-2229-10-143)
Supplement: Additional file 6 — Materials used for correlation analysis. This is a table. It shows the lines used for correlation analysis between the expression levels of ZmGW2-CHR4 and ZmGW2-CHR5 and the four yield-related traits. [file 1471-2229-10-143-S6.DOC]

### Additional file 6 – Materials used for correlation analysis

| Taxa | Heterotic group^a^ | Taxa | Heterotic group^a^ | Taxa | Heterotic group^a^ |
| --- | --- | --- | --- | --- | --- |
| 4F1 | Lancaster | K14 | Reid | P138 | Temp-tropic |
| Ji63 | Lancaster | Tie7922 | Reid | SW1611 | Temp-tropic |
| Mo17 | Lancaster | Ye107 | Reid | S37 | Temp-tropic |
| Ye515 | Lancaster | Ye478 | Reid | Si434 | Zi330 |
| ZaC546 | Lancaster | Ye488 | Reid | Zong31 | Zi330 |
| C8605 | Reid | Zheng58 | Reid | Cheng698 | Zi330 |
| Chang3 | Reid | Chang7-2 | TangSPT | Hai268 | Zi330 |
| U8112 | Reid | HZS | TangSPT | LK11 | Zi330 |
| 832 | Reid | Ji853 | TangSPT | Zi330 | Zi330 |
| B73 | Reid | Q1261 | TangSPT | 8902 | UN |
| Hu803 | Reid | Xi502 | TangSPT | 81162 | UN |
| HuangC | Reid | 87-1 | Temp-tropic | K22 | UN |
| J4112 | Reid | BT1 | Temp-tropic | LX9801 | UN |
| K10 | Reid | Dan599 | Temp-tropic |  |  |

UN, unknown

^a^ From Teng et al. [66]
